# Mechanisms underpinning natural variation in non-photochemical quenching kinetics

**DOI:** 10.1042/BST20253087

**Published:** 2025-09-18

**Authors:** Katarzyna Glowacka

**Affiliations:** 1 Department of Biochemistry and Center for Plant Science Innovation, University of Nebraska-Lincoln, Lincoln, NE, U.S.A.; 2 Institute of Plant Genetics, Polish Academy of Sciences, Poznań, 60-479, Poland

**Keywords:** natural variation, non-photochemical quenching, PsbS, VDE, xanthophyll cycle pigments, ZEP

## Abstract

Plants use light as an energy source to reduce carbon dioxide into carbohydrates during photosynthesis. However, when the incident light exceeds the photosynthesis rate, the excess energy must be dispersed, or it can result in the unregulated formation of harmful reactive oxygen species, especially in plants exposed to very high light or abiotic stress conditions that compromise photosynthetic efficiency. The excess energy is typically dispersed harmlessly as heat, which can be measured as non-photochemical quenching (NPQ) of chlorophyll fluorescence. NPQ kinetics vary within plant populations, and understanding the basis of this variation will contribute to improving resiliency to abiotic stresses, including high light, in crops. Here it is reviewed how three key NPQ genes, *Photosystem II subunit S* (*PsbS*), *Violaxanthin de-epoxidase* (*VDE*), and *Zeaxanthin epoxidase* (*ZEP*), contribute to natural variation in NPQ kinetics. *PsbS* expression level is an important determinant of NPQ variation, whereas VDE and ZEP contribute to NPQ variation via post-translational regulation related to natural variation in many genes affecting these enzymes’ activity. Post-translational mechanisms that influence NPQ, including redox regulation via thioredoxins and regulation of ascorbate availability, thylakoid lumen pH, and violaxanthin accessibility are discussed. There are also addressed NPQ regulatory mechanisms beyond PsbS, ZEP, and VDE, including natural regulation of light accessibility, modulation of light harvesting, and feedback from the steps following light harvesting. Finally, how this knowledge can be harnessed to engineer more resilient crops is briefly summarized.

## Introduction

**Figure 1:**
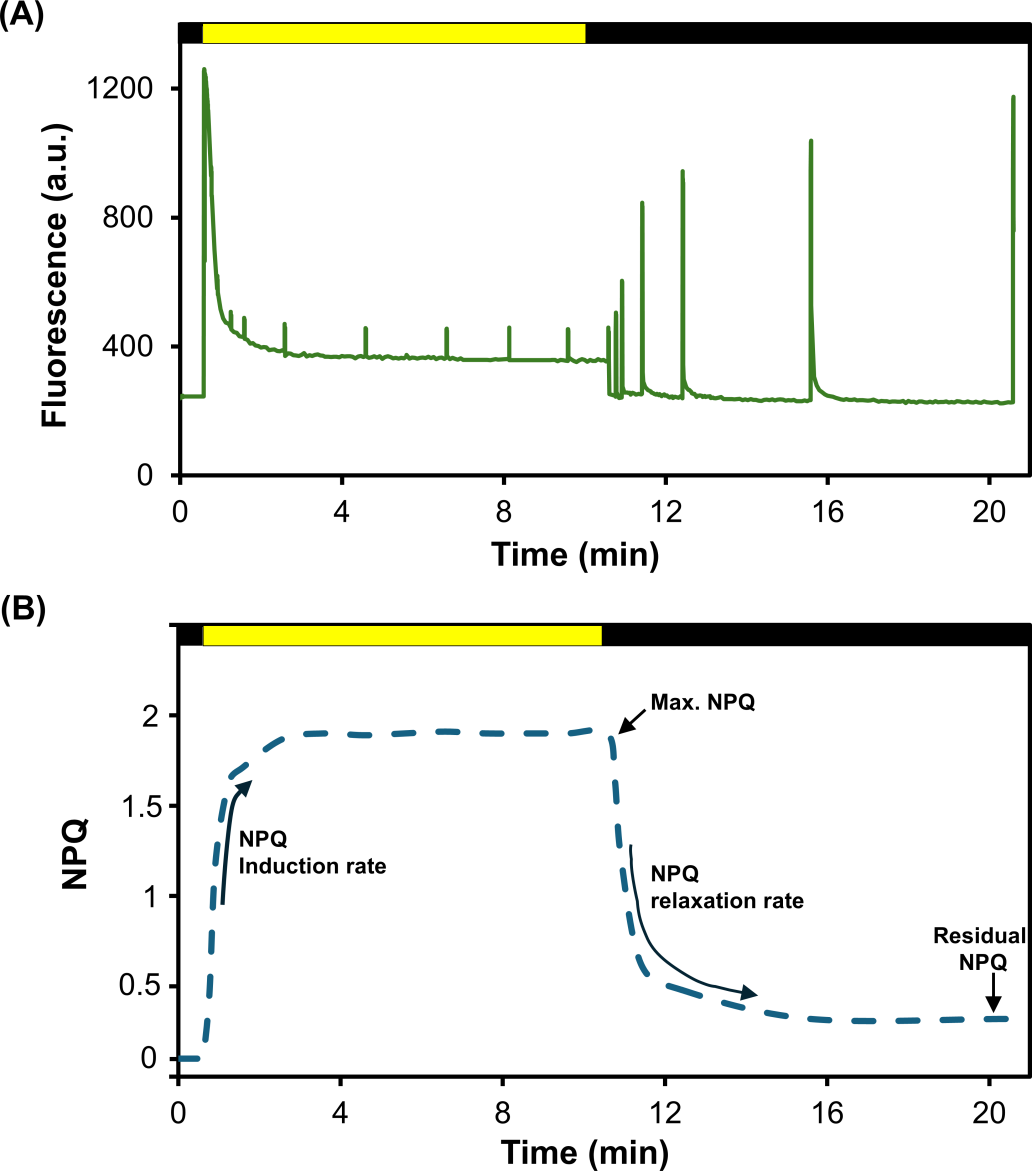
Response of NPQ kinetics to light and dark. (**A**) Representative pulse amplitude modulated (PAM) fluorescence quenching trace and (**B**) calculated NPQ induction and relaxation kinetics with four distinguished attributes. Measurements were taken on an Arabidopsis leaf. The yellow and black horizontal bars indicate light and dark treatments, respectively.

Under high-light stress conditions, light capture by plants exceeds the rate at which energy is utilized, resulting in reactive oxygen species (ROS) formation and photodamage [1–3]. Plants have evolved photoprotective mechanisms to dissipate excess light energy [4]. Rapid thermal dissipation of excess energy can be assessed by measuring the non-photochemical quenching (NPQ) of chlorophyll fluorescence [5] using pulse amplitude modulated fluorescence method (Figure 1). NPQ consists of multiple components with different rates of induction in the light and relaxation in the dark [6]. This review focuses on two of the fastest components of NPQ: energy-dependent quenching (qE) and zeaxanthin-dependent quenching (qZ). However, qE and qZ are not independent; consequential qE cannot be described independently of the slower NPQ components, such as qZ [7]. In light, NPQ kinetics can be divided into attributes such as NPQ induction rate and maximum NPQ (Figure 1B). Both are shaped by qE and qZ. In the dark, NPQ relaxation rate and minimum NPQ can be defined. Fast relaxing qE would mainly explain the rate of NPQ relaxation, and qZ would contribute mostly to the NPQ not relaxed after a short dark period, which is named a residual NPQ.

**Figure 2:**
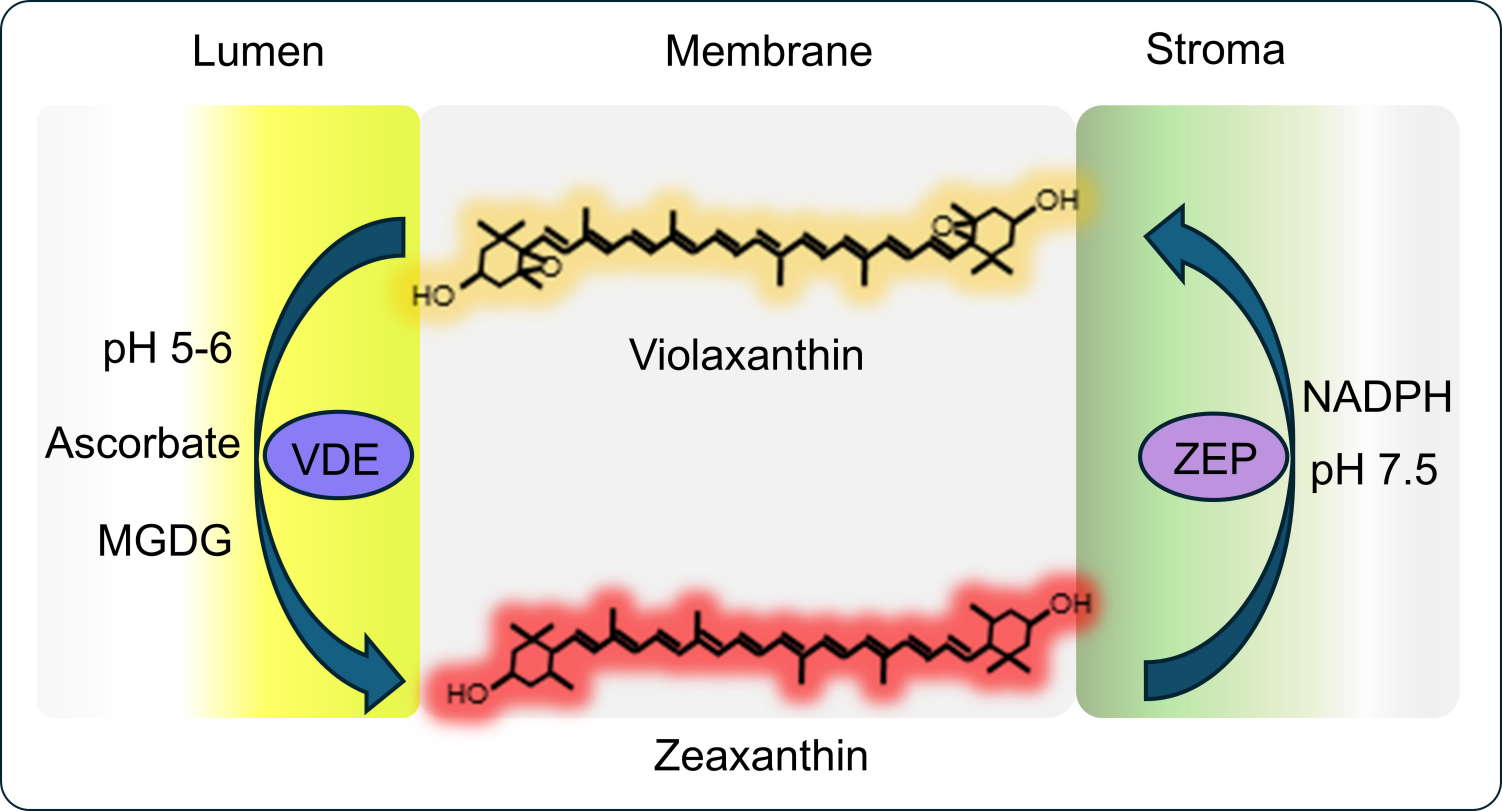
General model of the xanthophyll cycle operation. MGDG, monogalactosyldiacylglycerol glycerol; NADPH, nicotinamide adenine dinucleotide; VDE, violaxanthin de-epoxidase; ZEP, zeaxanthin epoxidase.

Pigments and proteins in thylakoid membranes of chloroplasts are organized into antennas of light-harvesting complexes (LHCs) that absorb light. The energy of absorbed light is used to excite chlorophylls in the photosystem II (PSII) reaction center, which extracts electrons from water that are then transferred through an electron transfer chain to photosystem I. Light-induced electron transport across the thylakoid membrane also powers the proton movement across thylakoid membranes to create a proton gradient lowering the pH in the lumen of thylakoids. For NPQ to occur, the photosynthetic antennas must switch from funneling energy into the reaction center to dispersing excess energy. This rearranging switch is stimulated by (1) buildup of the proton gradient across the thylakoid membrane, (2) protonation of PSII subunit S [photosystem II subunit S (PsbS)], and (3) conversion of the xanthophyll pigments violaxanthin (Vx) to zeaxanthin (Zx) via the intermediate antheraxanthin violaxanthin-antheraxanthin-zeaxanthin (VAZ) cycle, catalyzed by violaxanthin de-epoxidase (VDE) (Figure 2, 8–10). PsbS functions as a pH sensor that links NPQ to the trans-thylakoid pH gradient (ΔpH). PsbS catalyzes qE, and Zx is its allosteric regulator. The major LHCII contains the quencher for NPQ (reviewed in [3]), likely the xanthophyll pigment lutein [11]. When the thylakoid lumen has a low pH, PsbS dimers dissociate into monomers that bind LHCII, which might make it more sensitive to ΔpH [12]. This greater sensitivity of LHCII to ΔpH suggests changes in LHCII conformation leading to the flattening of the LHCII complex in the membrane and rearrangement of surrounding lipids [13–15]. The resulting hydrophobic mismatch in the thylakoid membrane between LHCII and lipids is thought to induce quenching in nearby LHCII trimers lacking PsbS [12]. The evidence also exists that Zx might allosterically control the interactions between PsbS and minor antenna proteins (Lhcb4 (CP29), Lhcb5 (CP26), and Lhcb6 (CP24)) needed to fully rearrange PSII–LHCII supercomplexes and make NPQ more responsive to low pH [16–18]. Under low-light conditions, zeaxanthin epoxidase (ZEP) converts Zx back to Vx to limit photoprotection and achieve more efficient energy utilization (Figure 2). Under various abiotic stress conditions and in many plant species, including the C_4_ grass *Miscanthus*, xanthophyll de-epoxidation also occurs in darkness [19,20].

The importance of NPQ is underscored by the phenotypes of plants lacking PsbS, VDE, or ZEP. Loss of PsbS raises ROS production under high-light conditions [21], prevents high-light acclimation [21], slows growth in fluctuating light environments [22], and diminishes plant fitness [23] and survival [22]. Loss of VDE blocks Zx formation and increases PSII damage [24–26] and lipid peroxidation [24]. Zx accumulation in plants lacking ZEP leads to higher NPQ values at low and moderate light intensities, faster induction of NPQ, and slower relaxation relative to wildtype [25–27], which slows growth under low-light conditions [28]. However, the more complex role of PsbS and Zx in the regulation of NPQ kinetics under a changeable light regime was also suggested [7]. Surprisingly, though, *PsbS*-silenced lines of tobacco (*Nicotiana tabacum*) with clear impartment of NPQ showed only a 22% drop in dry weight when grown in the field [29].

NPQ dissipates energy in high light, but plants incur a potential energy cost upon return to lower light. Indeed, faster relaxation of NPQ is associated with more biomass accumulation across accessions of African rice (*Oryza glaberrima*) [30]. Furthermore, accelerating recovery from photoprotection via a transgenic approach improved photosynthetic efficiency and growth in multiple crops [31–34]. However, there have been contrasting results in Arabidopsis [35] and potato [36]. Modulation of chloroplast-derived signals for stomatal opening via NPQ up-regulation in the PsbS-overexpressing lines also improved water use efficiency in tobacco [29,37]. Whether and how natural variation affects NPQ has received much less attention than the ecological context of NPQ and its biophysical, environmental, and molecular regulation (for recent reviews, see 3–5,19,38,39). Recently proposed semi-high-throughput methods of measuring NPQ kinetics, in combination with genomic data [40,41], have accelerated the identification of candidate genes associated with varied NPQ kinetics.

**Figure 3:**
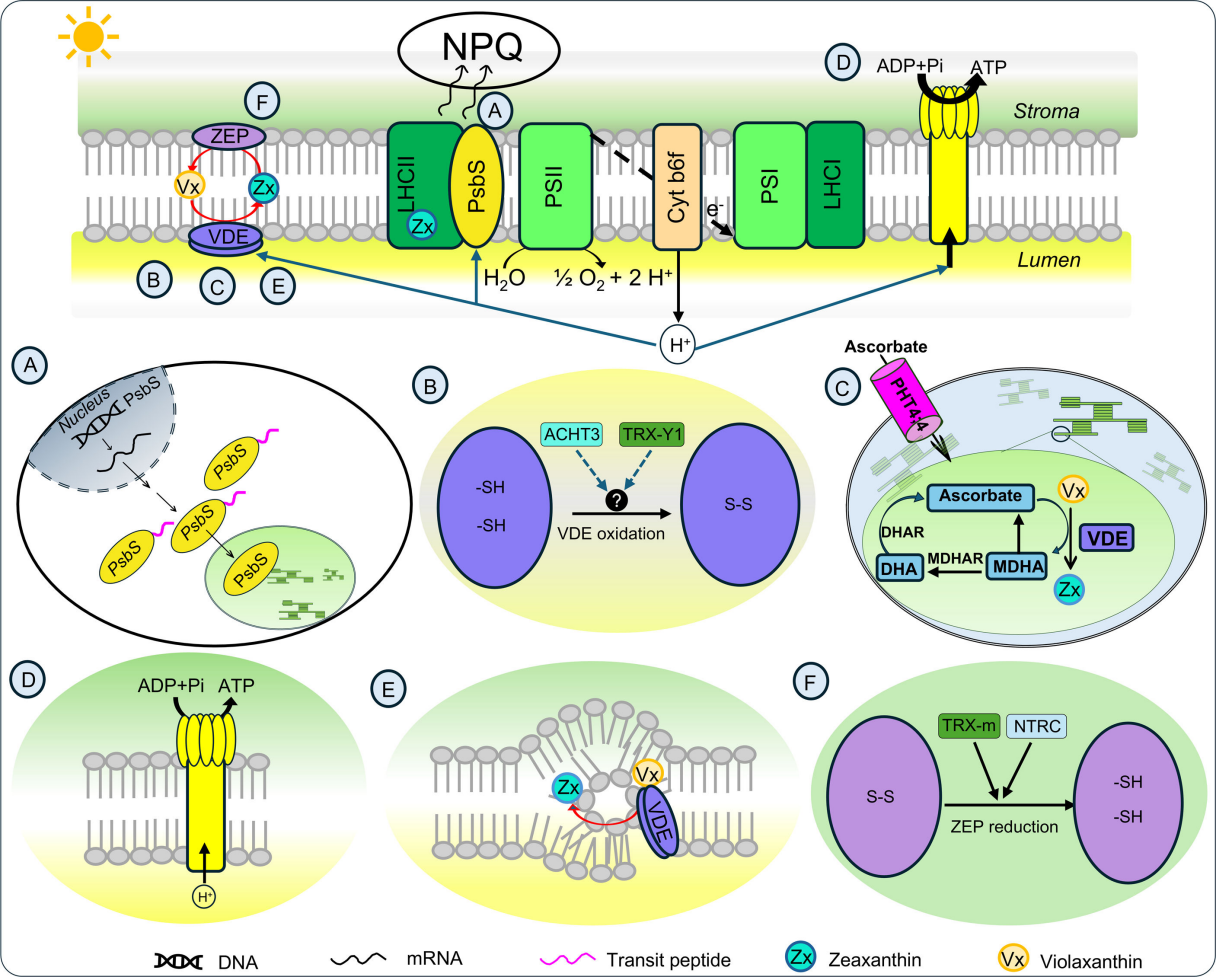
Possible routes of variation in NPQ kinetics involved photosystem II subunit S (PsbS), violaxanthin de-epoxidase, and zeaxanthin epoxidase. (**A**) Transcriptional regulation of *PsbS*. (**B**) Indirect regulation of VDE redox status, possibly by atypical Cys/His-rich thioredoxin 3 (ACHT3) and/or thioredoxin Y1 (TRX-Y1) that can reduce cysteine residues of unknown enzyme that is then oxidizing VDE. (**C**) Accessibility of the VDE cofactor ascorbate via expression of chloroplast ascorbate transporters (inorganic phosphate transporter 4;4 [PHT4;4]) and regeneration of a reduced form of ascorbate from monodehydroascorbate (MDHA) and dehydroascorbate (DHA). (**D**) Down-regulation of chloroplast adenosine triphosphatase activity contributing to lumenal acidification. (**E**) Regulation of VDE activity via the formation of inverted hexagonal phase domains in the thylakoid membrane. (**F**) Regulation of ZEP stability/activity, possibly via reduction of disulfide bonds by thioredoxin m (TRX-m) and/or NADPH thioredoxin reductase C (NTRC). ADP, adenosine diphosphate; ATP, adenosine triphosphate; Cyt b6f, cytochrome b6f complex; DHAR, dehydroascorbate reductase; H^+^, proton; LHCI, light-harvesting complexe I; LHCII, light-harvesting complexe II; MDHAR, monodehydroascorbate reductase; P_i_, inorganic phosphate; PSI, photosystem I; PSII, photosystem II; -SH, sulfhydryl group; S-S, disulfide bond; Vx, violaxanthin; Zx, zeaxanthin.

Considering the importance of NPQ for plant fitness in changeable environments and the opportunities to improve crops via manipulation of NPQ, this article reviews our emerging understanding of the mechanisms of natural variation in NPQ (Figure 3).

### PsbS as a major determinant of variation in NPQ

Genome-wide association studies (GWASs) of NPQ have shown that variation in the *PsbS* promoter is a major determinant for the natural variation of NPQ capacity in multiple species 41–48, Figure 3A). For instance, in rice (*Oryza sativa*), an insertion in the *PsbS* promoter region increases its expression levels in some accessions, leading to higher NPQ [46]. In maize (*Zea mays*), a single-nucleotide polymorphism (SNP) within a *PsbS* intron contributes to variation in NPQ, together with a *cis*-expression quantitative trait locus that colocalizes with *PsbS*; in addition, *PsbS* transcript levels were associated with NPQ in a transcriptome-wide association study [41]. Modulation of PsbS protein content via genetic modification in tobacco and Arabidopsis (*Arabidopsis thaliana*) showed that NPQ increased proportionally with PsbS [9,29,37]. An up-regulation of *PsbS* transcript levels was associated with higher NPQ values in response to high-light conditions, alone or in combination with other stresses, in some studies [46,49]. *PsbS* transcript levels are up-regulated in response to light and follow a diurnal pattern [9,50], possibly requiring the basic leucine transcription factor bZIP18 for light-dependent regulation of *PsbS* expression in Arabidopsis [51]. Notably, natural variation in the NPQ response to coastal late fall conditions (relatively lower light intensity in combination with chilling temperatures) in 284 Arabidopsis ecotypes was related to an SNP located in the *PsbS* promoter, with higher PsbS abundance being associated with higher NPQ [44].

### VDE and ZEP as determinants of NPQ variation

Two of the main sources of natural variation could either be transcriptional or post-translation regulation. One GWAS identified *VDE* [52] as a candidate gene associated with variation in NPQ, and none returned *ZEP* as a candidate [23,41,44,45], suggesting that the *ZEP* and *VDE* loci, including their promoter regions, lack sufficient variation for a significant association with variation in NPQ. In agreement, a GWAS for carotenoid-related traits using 155 maize inbred lines failed to detect any polymorphism in *ZmVDE1* that was significantly associated with these traits [53]. Moreover, there was no detectable correlation between polymorphisms in *ZmVDE1* and its transcript abundance. Notably, a comparison of the *ZmVDE1* sequence between domesticated maize and its wild relatives *Z. mays* ssp. *parviglumis* and *Z. mays* ssp. *mexicana* (teosinte) identified a potential sweep across *ZmVDE1* that severely diminished its nucleotide diversity. This suggests that the natural variation in NPQ related to differences in VDE and ZEP activity involves post-translational, rather than transcriptional, regulation of *VDE* and *ZEP*. In line with this hypothesis, VDE activity is regulated predominantly via post-transcriptional mechanisms during the acclimation of Arabidopsis plants to high-light conditions, despite diurnal changes in *VDE* expression levels [54]. Similarly, *ZEP* expression levels follow a diurnal pattern in multiple species, although they were unaffected by light intensity [54,55]. Experiments overexpressing *ZEP* or *VDE* in Arabidopsis suggested that ZEP controls the xanthophyll interconversion rate, whereas VDE had a modest effect [56]. Notably, VDE proteins from Arabidopsis, tobacco, and lettuce (*Lactuca sativa*) only differ by 9% of amino acids [57]. In agreement with this observation, GWAS in diverse plant species detected multiple candidate genes with potential roles in the post-transcriptional regulation of VDE and ZEP, as explored in more detail below [23,41,52].

For example, two thioredoxins are associated with variation in NPQ kinetics in maize [41]. Numerous chloroplast lumen proteins, including VDE, are targets of thioredoxin redox regulation [58] through the formation of reversible disulfide bonds between the thiol groups of two cysteine residues, which modulates protein activity, folding, and stability. VDE activity requires an oxidizing environment, which is promoted by the photosynthetic light reactions [38] and allows the formation of disulfide bonds for a more rigid structure, improved thermal stability, and catalysis [59]. Monomeric VDE contains 13 conserved cysteines, 12 of which form disulfide bonds. Although the proteins controlling VDE disulfide bond formation have yet to be determined, GWAS identified *Thioredoxin Y1* (*TRX-Y1*) polymorphisms as being strongly associated with the speed of PSII operating efficiency (ΦPSII) recovery in darkness, which is negatively correlated with NPQ relaxation in darkness; in addition, *atypical Cys His-rich thioredoxin 3* (*ACHT3*) was strongly associated with multiple NPQ traits, especially maximum NPQ in the light [41]. The functions and roles of ACHT3 and TRX-Y1 are not fully understood [60,61], but their absence in Arabidopsis impaired NPQ kinetics and might contribute to the redox regulation of VDE in the light (Figure 3B). Stroma-localized TRX-Y1 may promote the reduction in peroxiredoxin Q, which may sense the redox state of chloroplasts [62]. Experimental evidence suggests that VDE in the highly chilling-tolerant *Miscanthus sacchariflorus* is less sensitive to a reducing environment than that of the poorly chilling-tolerant *Miscanthus sinensis* [20].

Critical to VDE activity is its cofactor ascorbate, which is converted to its oxidized forms, monodehydroascorbate and dehydroascorbate (DHA), during the VDE catalytic cycle [63] (Figure 3C). The enzymes contributing to ascorbate recycling include monodehydroascorbate reductase (MDHAR) and dehydroascorbate reductase (DHAR). Ascorbate regeneration maintains a pool of reduced ascorbate via ascorbate regeneration; ascorbate levels are positively correlated with NPQ [64]. In C_4_-type grasses with strong chilling tolerance, the rate of NPQ induction rises in response to chilling and is correlated with DHA accumulation, suggesting that a higher abundance of the VDE cofactor in its oxidized form might reflect the post-translational up-regulation of VDE activity by cold exposure [20]. *MDHAR* or *DHAR* overexpression enhanced the tolerance of multiple plant species including tobacco, Arabidopsis, and potato (*Solanum tuberosum*) to abiotic stress conditions such as drought, cold, and heat (as reviewed in [65]. A GWAS in *M. sacchariflorus* identified *MDHAR*, encoding the peroxisome/cytosol-located MDHAR enzyme, as being associated with traits related to NPQ [66]. The same study identified *l-galactono-1,4-lactone dehydrogenase*, encoding an ascorbate biosynthetic enzyme, as associated with NPQ induction rate in the light. Notably, TRX-Y1 increases chloroplast MDHAR activity in Arabidopsis and might control reduction in the ascorbate pool [67]; indeed, a insertional mutant of *TRX-Y1* had impaired NPQ kinetics [41]. An ascorbate transporter, located in the chloroplast envelope and belonging to the phosphate transporter 4 family (PHT4;4), is important for NPQ induction in response to high light in Arabidopsis [68] (Figure 3C). Notably, *PHT4;4* was among the candidate genes in a study on the response of Arabidopsis ecotypes to abiotic stresses [69].

VDE is active in the low pH environment of the thylakoid lumen; therefore, modulation of the pH in the thylakoid lumen indirectly regulates VDE activity [38]. It has been speculated that VDE activation is driven by its dimerization with the help of protonation of four conserved amino acids, where the active VDE tightly binds to the thylakoid membrane. The lumenal pH is regulated not only by influx of protons via electron transport across the thylakoid membranes but also by efflux of protons with the help of the chloroplast ATP synthase. Therefore, down-regulation of ATP synthase conductivity to protons in response to slower ATP consumption contributes to higher light-driven proton-motive force, resulting in greater NPQ via activation of PsbS and VDE (Figure 3D). In line with this idea, NPQ variation across rice accessions was associated with polymorphisms in *ATP synthase gamma chain*, which could affect NPQ by contributing to proton gradient buildup [23]. In Arabidopsis, the trans-thylakoid proton gradient is controlled by multiple potassium (K^+^) and chloride (Cl^−^) channels in the thylakoid membrane [70,71]. An SNP within an intron of a cation efflux family gene in *Miscanthus* was associated with natural variation in the ΦPSII/NPQ ratio during photosynthetic efficiency recovery following cold exposure [66]. The ΦPSII/NPQ ratio is a measurement of the trade-off between ΦPSII being recovered and NPQ not relaxing after 12 min of darkness preceded by 10 min of light. Similarly, natural variation in a gene encoding the cation-selective high-conductance channel outer envelope protein 37 (OEP37) in maize was associated with ΦPSII recovery in darkness, which is inversely correlated with NPQ relaxation in darkness [41]. The Arabidopsis *oep37* mutant is severely impaired in multiple NPQ traits [41]. In principle, ion channels in the outer chloroplast envelope might regulate the trans-thylakoid proton gradient.

Finally, VDE activity is modulated by Vx availability, which is controlled by the lipid composition of thylakoid membranes [38]. The release of Vx from the xanthophyll-binding site of an antenna protein might be a prerequisite for its conversion to Zx via VDE [38]. VDE activity takes place in inverted hexagonal phase domains within the thylakoid membrane, which form due to the presence of the galactolipid monogalactosyldiacylglycerol (MGDG) [38] (Figure 3E). In addition, lipid flux, lower digalactosyldiacylglycerol levels, and higher MGDG levels influence the transition of LHCII from its light-harvesting to its photoprotective quenched state. During this transition, the mechanical stability of LHCII against unfolding changes, as does the hydrostatic membrane pressure of the LHCII complex [72]. There was a strong association between the rate of NPQ relaxation in the dark and chloroplast-located lysophosphatidic acid acyltransferase enzymes crucial for maintaining lipid homeostasis [41,73]. Work in soybean (*Glycine max*) suggested that the Zx biosynthetic pathway influences NPQ via natural variation in the genes encoding enzymes involved in the carotenoid biosynthetic pathway, including a β-hydroxylase and a monooxygenase [52].

In contrast with VDE, much less is known about how ZEP is regulated. ZEP activity is strongly down-regulated by hydrogen peroxide, which would increase the speed of NPQ induction and could lead to more sustainable NPQ under high-light conditions [74]. Natural variation in NPQ kinetics under water limitation in wheat (*Triticum aestivum*) and soybean was also suggested to be related to changes in ZEP activity rather than *ZEP* transcript levels [75,76]. ZEP contains nine cysteine residues and was controlled by light in Arabidopsis via redox changes possibly involving NADPH thioredoxin reductase C [77] or thioredoxin m [78] (Figure 3F). Under stress conditions, ZEP abundance is down-regulated via degradation to augment Zx accumulation [79,80]. The differential down-regulation of ZEP activity in rice might contribute to natural variation in Zx accumulation under cold stress and the natural variation in NPQ under stress conditions [81]. In *M. sacchariflorus*, chilling exposure triggered the dark accumulation of Zx, concomitant with an absence of changes in *ZEP* transcript levels in response to chilling, although compromised ZEP stability might contribute to final down-regulation of ZEP activity [20].

## NPQ variation: beyond PsbS, ZEP, and VDE

### Regulation via light accessibility

Releasing excess energy via heat, observed as NPQ, is one potential road for photoprotection. NPQ rapidly dissipates excess light energy, and other possible photoprotective mechanisms are slower, including chloroplast movement and the biosynthesis of photoprotective pigments that absorb light before it reaches chloroplasts and the light-harvesting antennas. Variation in photoprotective mechanisms other than NPQ would indirectly regulate NPQ strength; likewise, the interplay among multiple photoprotective mechanisms would optimize light utilization. In agreement with this notion, two plant-speciﬁc proteins involved in chloroplast movement in response to blue light were identified in maize: plastid movement impaired 1 and chloroplast unusual positioning 1 [82,83]. Natural variation in their encoding genes was associated with variation in NPQ kinetics via ΦPSII [41]. Chloroplast movement to the anticlinal walls (light-avoidance position) confers general photoprotection, whereas more limited chloroplast movement enhances NPQ [84]. Similarly, the accumulation of anthocyanin pigments in the vacuole offers photoprotection through absorption of yellow, green, and blue light, particularly under abiotic stress [85], affecting the quantity and quality of light that reaches chloroplasts [86]. Plant cells can deploy anthocyanins as quickly as a few hours after stress onset [87,88]. Under changing seasonal conditions, high anthocyanin levels compensate for low NPQ, although anthocyanin accumulation alone cannot provide sufficient photoprotection [89]. For instance, in *M. sacchariflorus*, NPQ and anthocyanins work together, and genes involved in anthocyanin biosynthesis are associated with variation in the speed of NPQ relaxation in the dark [20,66].

### Regulation via light harvesting

NPQ may be affected by variation in genes involved in electron transport, PSII light-harvesting antenna structure, and rearrangements of LHCII that trigger NPQ. In agreement with this, NPQ variation in rice was shown to be controlled by variation in genes encoding a light-induced protein 1-like protein, a thylakoid lumenal protein, a PSII 10 kDa protein, and a protein with a plastocyanin-like domain [23]. Furthermore, quantitative trait loci mapping for NPQ kinetics in the multi-parent advanced generation inter-cross (MAGIC) maize population identified the PSII antenna protein CP24 as a contributor to NPQ variation in maize, with *CP24* expression and CP24 abundance positively associated with greater NPQ [90]. CP24 is a minor antenna protein positioned between LHCII trimers and the core complex and is critical for energy transfer from distal LHCII to the PSII core [91]. However, the question of whether the effect of CP24 on NPQ is direct or indirect remains open.

Under low nitrogen (N) stress, genotype-dependent differences in PSII antenna size might indirectly regulate NPQ kinetics in maize, as reflected by changes in the chlorophyll *a*/*b* ratio [92]. In a study of natural variation in NPQ kinetics in maize, the relative change in the chlorophyll *a*/*b* ratio and maximum NPQ values under low-N conditions were negatively correlated. Fewer PSII reaction centers with larger PSII antennas would lead to higher maximum NPQ values under low-N treatment, while relatively stable maximum NPQ would lead to no change in the ratio between reaction centers and antennas in PSII. Complementary work in sorghum (*Sorghum bicolor*) grown under high or low N identified candidate genes for variation in residual NPQ at the end of the dark period, including *Non-yellowing 1* (*NYE1*), which encodes a chlorophyll catabolism enzyme that degrades photosynthetic pigments in LHCs [45,93]. The two alleles of *NYE1* present in the sorghum diversity panel showed significant differences in photosynthetic pigment abundances. In addition, residual NPQ variation at the end of the dark period was associated with a putative ortholog of Arabidopsis *suppressor of variegation 3 (SVR3)*, encoding a chloroplast Type A translation elongation factor. The Arabidopsis *svr3* mutant has decreased chlorophyll contents [94]. Interestingly, lower LHCII abundance during acclimation to high light or a drop in chlorophyll *b* levels was speculated to increase the formation of inverted hexagonal phase domains in the thylakoid membrane, allowing more rapid NPQ via faster xanthophyll de-epoxidation [95].

### Feedback following light harvesting

The low efficiency of photoassimilate transport from leaves to sink organs might result in inhibitory feedback that increases NPQ to balance energy sources and its utilization [8]. Natural variation in sugar transporter genes homologous to Arabidopsis *early-responsive to dehydration 6* was associated with variation in NPQ in soybean [52]. NPQ is also indirectly regulated by photochemical quenching, which is affected by stomatal movement. In agreement with this idea, one candidate gene associated with NPQ variation in maize was related to Arabidopsis *protein kinase 1B* (*PK1B*) [90]. The Arabidopsis chloroplast protein PK1B mediates stomatal opening in response to light, as a mutant failed to open its stomata upon light exposure [96]. While changes to the redox balance of the photosynthetic electron transport chain trigger signaling cascades that can modify nuclear gene expression via retrograde signaling [45,58].

**Perspectives**

- Non-photochemical quenching (NPQ) is indispensable for plant fitness and survival in the environment. NPQ appears to not have been fully optimized in the major crop species, providing opportunities for breeding and engineering better NPQ kinetics for greater photosynthetic efficiency, growth, and yield.
- Recent advances in semi-high-throughput approaches for investigating NPQ kinetics open the doors to conducting genome-wide association studies for the identification of genomic regions that shape variation in NPQ. Here, two possible sources of natural variation in NPQ, transcriptional regulation of photosystem II subunit S via polymorphisms in its promoter region and post-translational regulation of violaxanthin de-epoxidase and zeaxanthin epoxidase are outlined.
- How newly identified genes control NPQ is an important open question. In addition, variation in NPQ kinetics is regulated by environmental factors, including abiotic stresses, but the mechanisms have yet to be discovered. For instance, the dark regulation of NPQ (reviewed in [19], an example of acclimation to challenging environments, offers a promising avenue for developing more resilient crops to environmental extremes [20], although a deeper understanding of this phenomenon must be gained first.

## Abbreviations

ACHT3: atypical Cys/His-rich thioredoxin 3
ATP: Adenosine triphosphate
Cyt b6f: cytochrome b6f complex
DHA: dehydroascorbate
DHAR: dehydroascorbate reductase
GWASs: genome-wide association studies
LHCs: light-harvesting complexes
Lhcb4 (CP29): minor chlorophyll binding protein 4
Lhcb5 (CP26): minor chlrorophyll binding protein 5
Lhcb6 (CP24): minor chlorophyll binding protein 6
MAGIC: multi-parent advanced generation inter-cross
MDHA: monodehydroascorbate
MDHAR: monodehydroascorbate reductase
MGDG: monogalactosyldiacylglycerol
NADPH: nicotinamide adenine dinucleotide phosphate
NPQ: non-photochemical quenching
NTRC: NADPH thioredoxin reductase C
NYE1: Non-yellowing 1
OEP37: outer envelope protein 37
PAM: pulse amplitude modulated
PHT4;4: inorganic phosphate transporter 4;4
PK1B: protein kinase 1B
PSI: photosystem I
PSII: photosystem II
P_i_: inorganic phosphate
PsbS: photosystem II subunit S
ROS: reactive oxygen species
-SH: sulfhydryl group
SNP: single-nucleotide polymorphism
S-S: disulfide bond
SVR3: Suppressor of Variegation 3
TRX-Y1: thioredoxin Y1
TRX-m: thioredoxin m
VAZ cycle: violaxanthin-antheraxanthin-zeaxanthin cycle
VDE: violaxanthin de-epoxidase
Vx: violaxanthin
ZEP: zeaxanthin epoxidase
Zx: zeaxanthin
qE: energy-dependent quenching
qZ: zeaxanthin-dependent quenching
ΔpH: trans-thylakoid pH gradient
ΦPSII: PSII operating efficiency
